# Mind + Machine: ChatGPT as a Basic Clinical Decisions Support Tool

**DOI:** 10.7759/cureus.43690

**Published:** 2023-08-18

**Authors:** Marc Ayoub, Ahmad A Ballout, Rosana A Zayek, Noel F Ayoub

**Affiliations:** 1 Neurocritical Care, Northwell, Northshore University Hospital, Manhasset, USA; 2 Internal Medicine, Elmhurst Hospital Center, Mount Sinai School of Medicine, New York, USA; 3 Neurology, Donald and Barbara Zucker School of Medicine at Hofstra/Northwell, Long Island, USA; 4 Internal Medicine, Torrance Memorial Medical Center, Torrance, USA; 5 Otolaryngology-Head and Neck Surgery, Stanford Health Care, Palo Alto, USA

**Keywords:** large language model, generative artificial intelligence, triage, clinical decision support system, artificial intelligence in healthcare, chatgpt

## Abstract

Background

Generative artificial intelligence (AI) has integrated into various industries as it has demonstrated enormous potential in automating elaborate processes and enhancing complex decision-making. The ability of these chatbots to critically triage, diagnose, and manage complex medical conditions, remains unknown and requires further research.

Objective

This cross-sectional study sought to quantitatively analyze the appropriateness of ChatGPT (OpenAI, San Francisco, CA, US) in its ability to triage, synthesize differential diagnoses, and generate treatment plans for nine diverse but common clinical scenarios.

Methods

Various common clinical scenarios were developed. Each was input into ChatGPT, and the chatbot was asked to develop diagnostic and treatment plans. Five practicing physicians independently scored ChatGPT’s responses to the clinical scenarios.

Results

The average overall score for the triage ranking was 4.2 (SD 0.7). The lowest overall score was for the completeness of the differential diagnosis at 4.1 (0.5). The highest overall scores were seen with the accuracy of the differential diagnosis, initial treatment plan, and overall usefulness of the response (all with an average score of 4.4). Variance among physician scores ranged from 0.24 for accuracy of the differential diagnosis to 0.49 for appropriateness of triage ranking.

Discussion

ChatGPT has the potential to augment clinical decision-making. More extensive research, however, is needed to ensure accuracy and appropriate recommendations are provided.

## Introduction

The release of generative pre-trained transformer (GPT) chatbots, such as Open AI’s ChatGPT (San Francisco, CA, US) or Google’s Bard (Mountain View, CA, US), has led to the integration of generative artificial intelligence (AI) across many industries, including healthcare. [1] Recent studies have shown GPT’s potential in answering United States Medical Licensing Exam questions, responding to preventive medicine questions, and outperforming physicians in empathy [2-5]. Despite these rapid developments, the reliability of using GPT technology to quickly triage and initiate treatment plans for multiple simultaneous clinical scenarios remains understudied.

This cross-sectional study sought to quantitatively analyze the appropriateness of ChatGPT’s ability to triage, synthesize differential diagnoses, and generate treatment plans for nine diverse but common clinical scenarios. 

## Materials and methods

The Stanford Institutional Review Board (IRB) deemed this study exempt from review. Three overarching clinical presentations that represented common scenarios within cardiology, pulmonology, and neurology were chosen (Appendix, Table 1).

**Table 1:** Physician grading of ChatGPT responses Prompt 1: An eight-year-old boy with stridor; Prompt 2: An eight-year-old boy with wheezing; Prompt 3: An eight-year-old boy with barking cough; Prompt 4: A 52-year-old male with headache, altered mental status, and fever; Prompt 5: A 52-year-old female with headache, slurred speech, and facial droop; Prompt 6: A 52-year-old female with headache and visual auras; Prompt 7: A 72-year-old male with chest pain radiating to the left arm, diaphoresis, and shortness of breath; Prompt 8: A 72-year-old male with abdominal distension and dyspepsia after a large meal; Prompt 9: A 72-year-old male with chest pain two days after lifting heavy boxes. Pain is worse with movement and with palpation of the chest wall. SD: standard deviation

	Urgency ranking	Differential diagnoses accuracy	Initial Diagnostic Plan	Differential diagnosis completeness	Initial treatment plan	Overall usefulness	Overall Evaluation
Prompt 1, mean (SD)	4.8 (0.5)	4.4 (0.6)	4.2 (0.8)	3.8 (0.5)	4.6 (0.6)	4.4 (0.6)	4.4 (0.6)
Prompt 2	4.2 (0.8)	4.4 (0.6)	4.2 (0.5)	4.2 (0.5)	4.4 (0.6)	4.4 (0.6)	4.2 (0.5)
Prompt 3	4.0 (0.7)	4.6 (0.6)	4.2 (0.5)	4.0 (0)	4.2 (0.8)	4.4 (0.6)	4.2 (0.8)
Prompt 4	4.2 (0.5)	4.2 (0.5)	3.8 (0.5)	4.2 (0.5)	4.0 (0)	4.0 (0)	4.0 (0)
Prompt 5	4.4 (06)	4.6 (0.6)	4.2 (0.5)	4.0 (0.7)	4.2 (0.5)	4.0 (0.7)	4.2 (0.5)
Prompt 6	4.6 (0.6)	4.4 (0.6)	4.2 (0.8)	4.2 (0.5)	4.8 (0.5)	4.6 (0.6)	4.6 (0.6)
Prompt 7	4.4 (0.6)	4.2 (0.5)	4.4 (0.6)	4.4 (0.6)	4.8 (0.5)	4.8 (0.5)	4.4 (0.6)
Prompt 8	4.2 (0.8)	4.2 (0.5)	4.4 (0.6)	4.2 (0.5)	4.6 (0.6)	4.6 (0.6)	4.2 (0.5)
Prompt 9	4.0 (1.2)	4.4 (0.6)	4.0 (0.7)	3.8 (0.8)	4.0 (0.7)	4.0 (0.7)	4.0 (0.7)
Overall	4.2 (0.7)	4.4 (0.5)	4.2 (0.6)	4.1 (0.5)	4.4 (0.6)	4.4 (0.6)	4.2 (0.5)

Three slightly variable scenarios were then developed for each clinical presentation that represented different most likely diagnoses with varying degrees of clinical severity. The prompts were developed through discussion among the authors and collective agreement regarding the specific wording, appropriateness of each scenario, and prevalence of presenting scenarios. Scenarios were intended to have some similar and overlapping symptoms to better assess ChatGPT's capabilities. Clinical scenarios were then input into ChatGPT-4 using the prompt: “You are a healthcare provider. You are presented with three patient scenarios. Your task is to triage and rank the most urgent patients, give a differential diagnosis for each patient, and provide the initial steps for their diagnostic and treatment plans.” All prompts and responses were in English. 

Grading was performed using a standardized five-point Likert scale of agreeability by five board-certified physicians of various subspecialty training: MA (internal medicine residency, board-certified in internal medicine, board-eligible in neurocritical care), AA (neurology residency, board-eligible in neurology); RZ (internal medicine residency, board-certified in internal medicine), CZ (internal medicine residency, board-certified in internal medicine, did not meet authorship criteria), and NA (otolaryngology residency, board-eligible in otolaryngology). All authors were blinded to each other’s responses. The ChatGPT prompts were scored based on the following: (1) appropriateness of the urgency ranking; (2) accuracy of the differential diagnosis; (3) completeness of the differential diagnosis; (4) completeness of the differential diagnosis; (5) overall usefulness of the response; and (6) overall evaluation. Scores ranged from 1 (entirely inappropriate, inaccurate, or incomplete) to 5 (entirely appropriate, accurate, or complete). The means, standard deviations, and variances were calculated, and graders were blinded to each other’s responses.

## Results

The overall evaluation of the nine clinical scenarios received a mean ± SD grade of 4.5 ± 0.5, ranging from 4.0 ± 0 to 4.6 ± 0.6. (Table 1) The mean ± SD grade was highest for ChatGPT's ability to generate accurate differential diagnosis (4.4 ± 0.5), initiate a treatment plan (4.4 ± 0.6), and its overall usefulness (4.4 ± 0.6), while the completeness of the differential diagnosis received the lowest grade (4.1 ± 0.5) (Table 1). The lowest variance in physician grading was for the accuracy of differential diagnosis generation (0.24) and the greatest for the appropriateness of urgency ranking (0.49) (Table 2). 

**Table 2:** Variance among healthcare provider scores of ChatGPT responses

	Urgency ranking	Differential diagnoses accuracy	Initial Diagnostic Plan	Differential diagnosis completeness	Initial treatment plan	Overall usefulness	Overall Evaluation
Variance	0.49	0.24	0.33	0.26	0.34	0.33	0.28

## Discussion

Generative AI has been touted as a major innovative force with many potential applications within and outside healthcare [1,3-6]. Despite the excitement, the medical community must first determine whether this technology is safe for healthcare. This study showed relatively high scores for ChatGPT’s responses to a variety of medical scenarios, and this was especially true for the differential diagnosis and initial treatment plan, but least so for the completeness of the differential diagnosis. Overall, this study shows that ChatGPT could potentially augment clinicians in their daily decision-making but cannot replace a clinician.

While the scores in this study were relatively high, clinicians must ask, "What is good enough?" Human medical errors now represent the third leading cause of death in the United States, and generative AI clinical decision support tools may help to remedy this issue [1,7]. When considering these tools, clinicians perhaps could reframe their evaluation based on whether the tools offer an improvement from the status quo rather than whether they are perfect. Notably, not all physicians scored the passages similarly in this study. The variance in this study emphasizes that, when analyzing generative AI responses, clinicians and researchers must consider differences in care among human providers and the possibility of multiple correct pathways.

There are some limitations to this study. Only nine scenarios were developed, and the inherent nuance of every clinical situation makes it difficult for even clinicians to agree on the most appropriate management plans for every patient scenario. The medico-legal ramifications of using these models also need further evaluation [8]. Additionally, AI models may provide slightly different answers based on the specific wording of the prompts. This phenomenon is termed "prompt engineering" and has become a topic of greater importance [9]. In order to maximize the benefit of AI models, users and clinicians must learn how to optimize the wording of the prompt used. This is a known limitation of ChatGPT analyses, but this study still demonstrates an important evaluation of ChatGPT's capabilities. Despite these limitations, this study sparks an important conversation about the evolving landscape of healthcare and the inevitable blend of human and machine expertise.

## Conclusions

ChatGPT and similar generative AI chatbots have the potential to augment clinical decision making. By challenging the boundaries between machine and human expertise, this study sparks an important conversation about the evolving landscape of healthcare. Additional research is required to ensure the safety of this technology in a variety of clinical scenarios and levels of urgency. 

## Appendices

Scenario 1: Airway/breathing

You are a healthcare provider. You are provided with three patient scenarios. Your task is to triage and rank the most urgent patients, give a differential diagnosis for each patient, and provide the initial steps in their diagnostic and treatment plans.

1. 8-year-old with stridor

2. 8-year-old with wheezing

3. 8-year-old with barking cough

Scenario 2: Headache

You are a healthcare provider. You are provided with three patient scenarios. Your task is to triage and rank the most urgent patients, give a differential diagnosis for each patient, and provide the initial steps in their diagnostic and treatment plans.

1. 52-year-old male presents with headache, altered mental status, and fever

2. 52-year-old male presents with headache, slurred speech, and facial droop

3. 52-year-old female presents with headaches and visual auras

Scenario 3: Chest pain

You are a healthcare provider. You are provided with three patient scenarios. Your task is to triage and rank the most urgent patients, give a differential diagnosis for each patient, and provide the initial steps in their diagnostic and treatment plans.

1. 72-year-old male with chest pain radiating to the left arm, diaphoresis, and shortness of breath.

2. 72-year-old male with abdominal distension and dyspepsia after a large meal

3. 72-year-old male with chest pain two days after lifting heavy boxes. The pain is worse with movement and palpation of the chest wall.
